# Trimethoprim-sulfamethoxazole-induced hyponatremia in an elderly lady with *Achromobacter xylosoxidans* pneumonia: Case report and insights into mechanism

**DOI:** 10.1097/MD.0000000000020746

**Published:** 2020-08-14

**Authors:** Michael George Zaki Ghali, Marc J. Kim

**Affiliations:** a Departments of Neurological Surgery, Internal Medicine, and Neurophysiology, Karolinska Institutet, Stockholm, Sweden; b Departments of Neurological Surgery, Neurophysiology, and Internal Medicine, University of Oslo, Oslo, Norway; c Departments of Neurological Surgery and Neurophysiology, University of Finland, Helsinki, Finland; d Department of Neurological Surgery, University Hospital Zurich, Zurich, Switzerland; e Department of Neurological Surgery, University of California, San Francisco, San Francisco, CA, USA; f Department of Neurological Surgery, Barrow Neurological Institute, Phoenix, AZ, USA; g Departments of Neurological Surgery and Internal Medicine, Johns Hopkins Medical Institute, Baltimore, MD, USA; h Department of Neurological Surgery, University of Toronto, Toronto, ON, Canada; i Department of Internal Medicine, University of Pennsylvania, 3400 Spruce Street, Philadelphia, PA.

**Keywords:** hyponatremia, mechanism, renal salt wasting, syndrome of inappropriate antidiuretic hormone, tolvaptan, trimethoprim-sulfamethoxazole

## Abstract

**Rationale:**

Hyponatremia occurs frequently in the hospital setting and may be attributable to a host of etiologies. Drugs are frequently implicated. Trimethoprim-sulfamethoxazole (TMP/SMX) represents a well-recognized pharmacologic precipitant of drug-induced hyponatremia, with several reports extant in the retrievable literature. Nephrologists thus debate the mechanisms giving rise to TMP/SMX-induced hyponatremia and the precise mechanism by which treatment with TMP/SMX generates reductions of serum sodium concentration remain controversial. The agent has a well-known effect of antagonizing the effects of aldosterone upon the distal nephron. Renal salt wasting and the syndrome of inappropriate antidiuretic hormone secretion represent implicated mechanistic intermediaries in TMP/SMX-induced hyponatremia.

**Patient concerns:**

The patient endorsed no explicit concerns.

**Diagnoses:**

We describe the case of an 83-year-old female clinically diagnosed with pneumonia found to have an initial serum sodium in the range of 130 to 134 mEq/L consistent with mild hyponatremia upon admission. Sputum cultures grew *Achromobacter xylosoxidans* susceptible to TMP/SMX. The patient's serum sodium concentration precipitously decline following institution of treatment with TMP/SMX to 112 to 114 mEq/L during the course of 5 days.

**Interventions:**

Severe hyponatremia proved recalcitrant to initial therapy with supplemental salt tabs and standard doses of the vasopressin receptor antagonist tolvaptan.

**Outcomes:**

Escalating doses of tolvaptan increased the patient's sodium to 120 to 124 mEq/L. The patient was transferred to another hospital for further management. During her stay, the patient did not exhibit frank or obvious clinical features consistent with hyponatremia nor readily appreciable evidence of volume depletion.

**Lessons:**

TMP/SMX represents a frequent, though underreported cause of hyponatremia in the hospital setting several authors believe natriuresis may represent the most common mechanism underlying TMP/SMX-induced hyponatremia. Evidence implicating natriuresis to be mechanistic in TMP/SMX-induced hyponatremia include clinically appreciable hypovolemia and resolution of hyponatremia with oral or intravenous salt repletion. Salt repletion failed to monotherapeutically enhance our patient's hyponatremiadisfavoring renal salt wasting as originately mechanistic. Contemporaneous refractoriness of serum sodium to fluid restriction nor standard doses of tolvaptan confounded our initial attempts to mechanistically attribute the patient's hyponatremia to a specific cause. Clinical euvolemia and rapid response of hyponatremia to exceptionally high doses of tolvaptan strongly favors syndrome of inappropriate antidiuretic hormone to represent the chief mechanism by which TMP/SMX exacerbates hyponatremia.

## 1 Introduction

Hyponatremia complicates the clinical course of approximately one-tenth of hospitalized patients^[1,2]^ and significantly enhances the risk of a patient prematurely expiring during the hospital course.^[3]^ Factors enhancing the risk of developing hyponatremia during the hospital course include elderly age, African-American ethnicity, reduced glomerular filtration rate or impaired tubular function,^[4]^ and active therapy with thiazide or loop diuretics, alternately correlative or causative.^[5–7]^ Authors patho-mechanistically classify reduction of serum sodium concentration into hypovolemic hyponatremic (ie, gastrointestinal or renal fluid-salt losses, salt wasting nephropathy, thiazide or loop diuretics; primary or secondary hypoaldosteronism), euvolemic hyponatremic (ie, syndrome of inappropriate antidiuretic hormone [SIADH] secretion, hypothyroidism, “beer potomania,” adrenocortical insufficiency, psychogenic polydipsia), and hypervolemic hyponatremic (ie, congestive heart failure, nephrotic syndrome, cirrhosis, acute and chronic kidney injury) etiologies (Table 1  ). A myriad of medications may purportedly precipitate hyponatremia through various mechanisms. Authors have described hyponatremia developing in patients receiving trimethoprim-sulfamethoxazole (TMP/SMX) therapy to treat *Pneumocystis jiroveci* pneumonia,^[8,9]^ nocardiosis,^[10,11]^ or urinary tract infection.^[12,13]^ Patients receiving higher doses of TMP/SMX develop biochemically evident hyponatremia with a much greater prevalence compared with individuals receiving lower doses of the compound.^[4]^ Renal dysfunction synergistically enhances the risk of developing aberrations of serum electrolyte concentrations in patients receiving TMP/SMX monotherapy.^[4]^ We detail our clinically salient experience with, and briefly review the literature evaluating, TMP/SMX-induced hyponatremia in the clinical setting, seeking to illumine the spectrum of mechanistic pathogenesis.

**Table 1:**
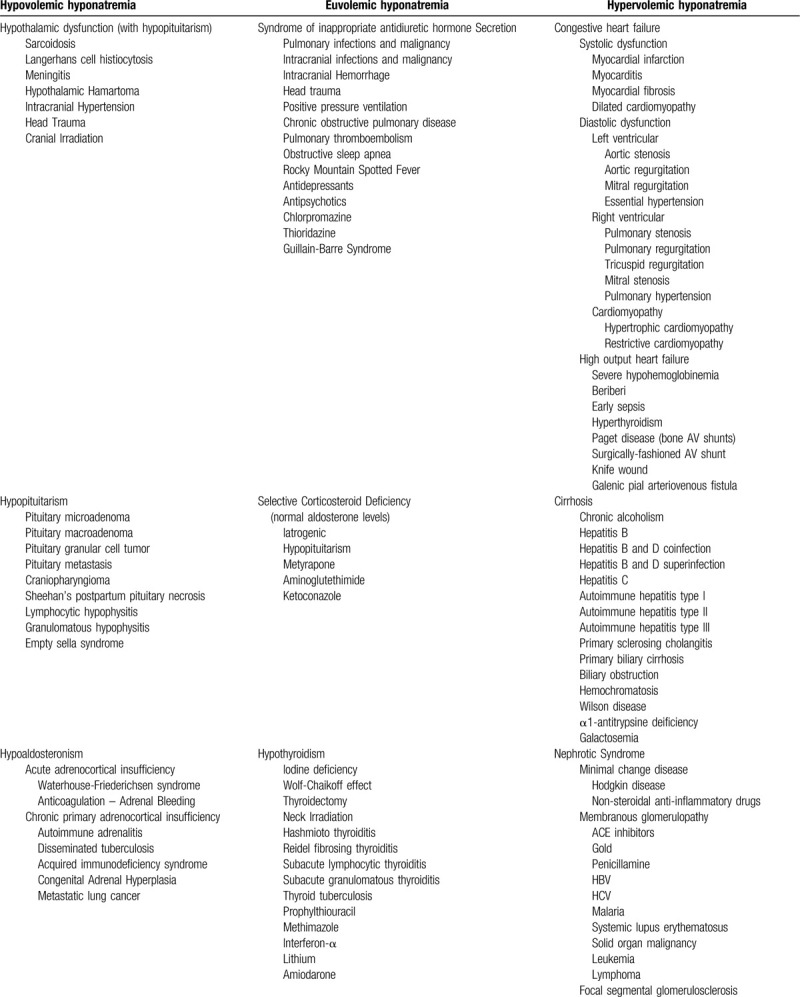
Abbreviated differential diagnosis of hyponatremia.

**Table 1 (Continued):**
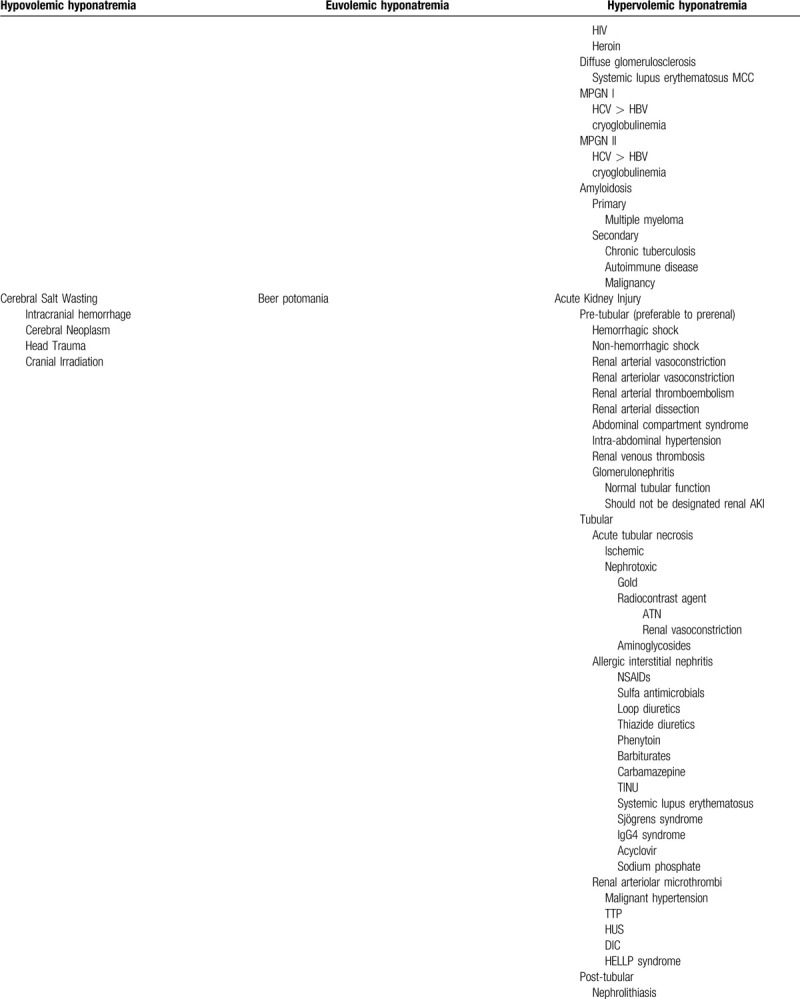
Abbreviated differential diagnosis of hyponatremia.

**Table 1 (Continued):**
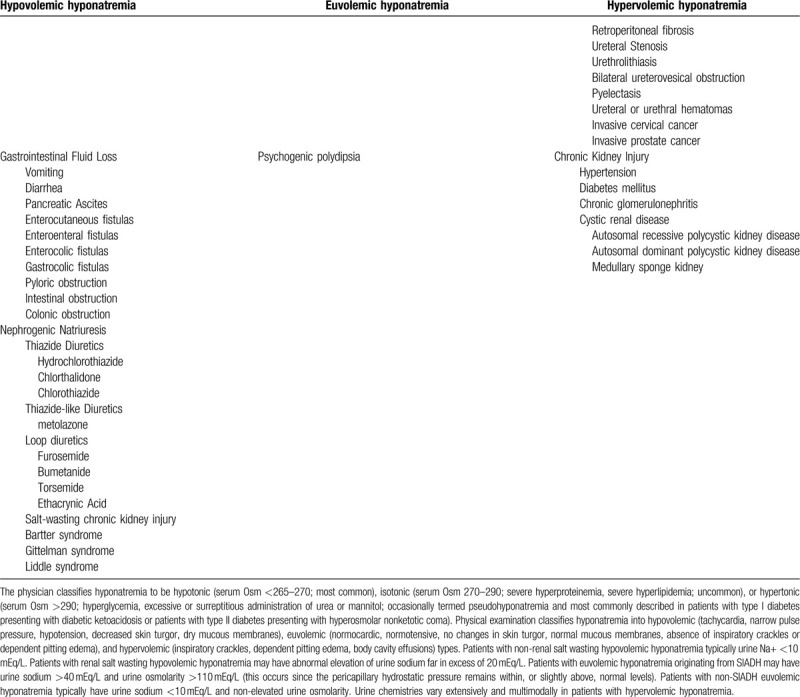
Abbreviated differential diagnosis of hyponatremia.

## 2 Methods

We report on an 83-year-old lady with *Achromabacter xylosoxidans* pneumonia, who developed severe worsening of hyponatremia following institution of TMP/SMX therapy. Prof. Dr. Med. M.G.Z. Ghali and Prof. Dr. Med. M. Kim performed a through, detailed, and meticulous evaluation of all clinical, laboratory, and imaging data. Ethical approval of this single case report by the institutional review board of the University of Oslo (September 1, 2016). We concurrently performed a comprehensive survey of the PubMed database for keywords and terms including “hyponatremia,” “etiology,” “diagnosis,” “treatment,” “trimethoprim-sulfamethoxazole,” “trimethoprim,” “sulfamethoxazole,” “TMP/SMX,” “medication,” “drug,” “mechanism,” “vasopressin,” “renal salt wasting,” “syndrome of inappropriate antidiuretic hormone secretion,” “renal,” and “hemodynamics” alone or in combination. Our search generated a plethora of articles. We perused these and selected clinical case reports and series of patients developing hyponatremia or experiencing reduction of serum sodium concentrations in the context of receiving TMP/SMX. We included cases in which treatment was evidently causative of the patient's hyponatremia and/or presented a thoughtful and considerate evaluate of putative mechanisms causing drug-induced hyponatremia.

## 3 Case description

An 83-year-old lady presented with chief complaints of “shortness of breath” and “nausea.” Roentgenographic evaluation of the chest revealed pulmonary infiltration. Culture samples obtained from induced sputum prolifically grew the organism *A xylosoxidans*. The pathogen proved resistant to several antibiotics. We obtained infectious disease consultation, who recommended prompt institution of TMP/SMX therapy to complete a 10 day course. We accordingly initiated TMP/SMX therapy, without much improvement to our chagrin and guarded disappointment. We immediately switched antimicrobial therapy to the broad spectrum carbapenem meropenem. Sensitivity of the pathogen to TMP/SMX led us to discontinue meropenem therapy and reinstitute with the originally administered agent. The patient continued to endorse copious mucus production, which was readily evident upon, and consistent with, our daily examination of the patient. We provided the patient inhaled hypertonic saline and respiratory physiotherapy in order to ameliorate her exuberant upper airway secretions and expedite her symptomatic convalescence. The patient exhibited no clinically appreciable bouts of acute respiratory distress during her otherwise uncomplicated hospital course, requiring only 2 to 3 L of supplemental oxygen by nasal cannula to treat mild decrements of the noninvasively determined arterial oxygen saturation.

Our more pressing clinical concern was the patient's steadily decrementing serum sodium levels, which, though biochemically significant, remained below the threshold of clinical detection. Before instituting TMP/SMX, the patient's serum sodium ranged between 130 and 134 mEq/L. We provided the patient with orally-administered TMP/SMX 4 times daily, which was continued for a duration of 4 days before providing the medication intravenously (TMP/SMX 160/800 mg) every 8 hours. We indicate correlation between the patient's serum sodium and TMP/SMX levels in Table 2. The patient received a total of 4 L of intravenously administered normal saline during the first 3 days of treatment with TMP/SMX. Serum sodium concentration continued to trend down daily, reaching a nadir of 110 mEq/L 4 days following institution of therapy. Serum osmolarity (242–273 mOsm/L) confirmed a hypotonic etiology of the patient's hyponatremia. Elevated urine sodium concentrations (117–152 mEq/L) and osmolarity (459–478 mOsm/L) led us to initially posit the SIADH secretion to be the culprit and chiefly mechanistic causing the patient's, putatively TMP/SMX-induced, hyponatremia. We accordingly provided the patient with salt tabs (a total of 12 g over 3 days) and instituted free water restriction. Measures which failed to appreciably enhance her serum sodium concentration. Uric acid and blood urea nitrogen concentrations of 2.2 mg/dL and 7 to 10 mg/dL, respectively, proved biochemically consistent with euvolemia or even slight hypervolemia. Low blood urea nitrogen may alternatively reflect old age, female gender, frailty, and a relatively low muscle mass.

**Table 2:**
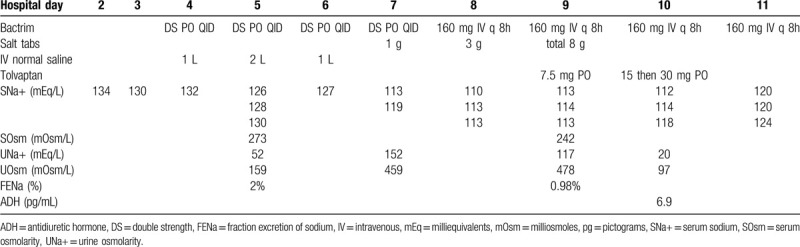
Laboratory data and course of hyponatremia.

Our bafflement led us to re-consult the nephrology service, who astutely recommended dose escalation of tolvaptan to 7.5 mg daily. Further dose escalation of tolvaptan to 15 mg daily proved necessary, though unfortunately failed to generate appreciable augmentation of her serum sodium concentrations, remaining between 112 and 114 mEq/L. Profound dose escalation of tolvaptan to 30 mg daily (in divided doses) successfully, though marginally, elicited increases of serum sodium concentration to approximately 120 mEq/L. Fractional excretion of sodium decreased from 2% to 0.98% following institution of tolvaptan therapy, indicating distal nephron responsivity to vasopressin receptor antagonism and implying functionality of antidiuretic hormone (ADH) receptors. The patient's serum ADH level was 6.9 pg/mL toward the end of her stay. The patient suffered from pestilent nausea, precluding the facile administration of medications via the oral route. The patient experienced a speedy convalescence from pneumonia during the next few days. However, due to previously receiving extensive care at an outside hospital, the patient's family and the patient herself accordingly requested transfer to her familiar institution.

## 4 Discussion

We describe the case of an 83-year-old lady presenting with pneumonia and mild hyponatremia. The patient initially presented with a serum sodium mildly below the statistically-derived 95% confidence interval typifying “normal.” Institution of TMP/SMX therapy precipitated a precipitous decline in her serum sodium concentration. Treatment with salt tabs, fluid restriction, and low to moderate doses of the vasopressin receptor antagonist tolvaptan, failed to generate appreciable increments of the serum sodium concentration. Extraordinarily high doses of tolvaptan finally generated increases of the patient's serum sodium concentration. Euvolemia, low serum concentration of blood urea nitrogen, and eventual responsivity to very high doses of tolvaptan supported the SIADH secretion to be the most likely culprit intermediary mechanism of TMP/SMX-induced hyponatremia, though we do not exclude renal salt wasting to be contributory. We present a brief review of the literature evaluating TMP/SMX-related hyponatremia, seeking to illumine the set of mechanisms which may generate clinical and subclinical reductions of the serum sodium

### 4.1 Hyponatremia in patients receiving TMP/SMX monotherapy for pulmonary infections

Authors have reported the development of hyponatremia in the context of patients receiving TMP/SMX to treat pulmonary infections. Herzog and colleagues (2016)^[10]^ describe a 66-year-old lady having previously undergone renal transplantation who developed Nocardiosis. TMP/SMX represented the presumptive etiology of de novo hyponatremia, requiring a change in therapy to amikacin and minocycline. Garner and colleagues (2017)^[11]^ describe a 57-year-old lady suffering from ulcerative colitis who developed disseminated nocardiosis while receiving infliximab and prednisone immunosuppressive therapy. The patient developed severe and symptomatic hyponatremia following initiation of TMP/SMX therapy, though, contrasted with the previous case, no change in treatment regimen was made and the patient was discharged on this medication to complete her antimicrobial course. Noto and colleagues (1995)^[14]^ describe 2 patients with *P jiroveci* pneumonia developing hyponatremia in the context of receiving TMP/SMX therapy, resolving with intravenous administration of salt-enriched fluids, without discontinuation of the medication. David and Ross (1998)^[8]^ similarly describe 2 patients with *P jiroveci* pneumonia, who developed severe hyponatremia following institution of treatment with high doses of TMP/SMX. Serum sodium concentrations increased in response to intravenous administration of salt-rich potassium-free fluid, implicating renal salt wasting to represent the most likely culprit intermediary mechanism. Babayev and colleagues (2013)^[9]^ describe a 28-year-old gentleman with human immunodeficiency virus (HIV) who developed *P jiroveci* pneumonia and a precipitous decrease in serum sodium from 135 mEq/L to 117 mEq/L following treatment with TMP/SMX, initially attributed to SIADH, though clinical evidence of hypovolemia, along with high renin and aldosterone levels, implicated renal salt wasting to represent the chief intermediary mechanism. The authors specifically postulated the hyponatremia to be caused by blockade of epithelial sodium channels by trimethoprim. Serum sodium concentrations expectedly increased to 126 to 128 mEq/L following discontinuation of TMP/SMX monotherapy. Precipitous declines in serum sodium in response to TMP/SMX in HIV patients^[9,15]^ may mechanistically reflect a reduced threshold to elicit secretion of ADH in response to serum osmolarity in the setting of infection with the human immunodeficiency virus.^[16,17]^ In stark contradistinction with the above case indicating renal salt wasting to represent the likely mechanism of TMP/SMX-induced hyponatremia, our patient's low serum sodium failed to respond adequately to salt tabs, fluid restriction, or traditionally-used doses of the vasopressin antagonist tolvaptan, though eventually responded somewhat to very high doses of the agent. Along with clinical euvolemia, low blood urea nitrogen concentration, and elevated levels of serum ADH corroborating exuberant elaboration of ADH to represent the most likely intermediary mechanism causing TMP/SMX-induced reductions of serum sodium concentration.

### 4.2 Hyponatremia in patients receiving TMP/SMX monotherapy for urinary tract infections

Chaitanya and colleagues (2015)^[12]^ describe a 49-year-old gentleman having previously undergone renal transplantation and actively receiving TMP/SMX to treat *Escherichia coli* urinary tract infection, whose clinical course was complicated by the development of hyponatremia. The patient exhibited severe volume depletion, tachycardia, hypotension, and severe hyponatremia, with a serum sodium concentration of at 115 mEq/L. Elevation of the UOsm (142 mOsm/L) and UNa (142 mEq/L), a fractional excretion of uric acid less than 10%; and resolution of hyponatremia with discontinuation of TMP/SMX and institution of a high salt diet (135 mEq/L after 4 days) evidenced renal salt wasting to represent the chief intermediary mechanism mediating drug-induced hyponatremia.^[18]^ Huntsberry and colleagues (2015)^[13]^ describe a case analogous to the one we present. An 82-year-old lady suffering from complicated cystitis presenting evidenced mild hyponatremia (132 mEq/L) upon admission and experienced a precipitous reduction in serum sodium concentration to 121 mEq/L following institution of a 7-day course of TMP/SMX monotherapy. Serum sodium concentration fortuitously recovered within the physiologically normal range within 1 week following successful completion of her antimicrobial course. The patient exhibited an analogous reduction in serum sodium concentration from 138 to 129 mEq/L when re-administered TMP/SMX to treat a subsequent episode of presumed cystitis. TMP/SMX monotherapy was halted in response to negative urine cultures, with serum sodium concentrations improving to 134 mEq/L. Decreases of the serum sodium concentration immediately following institution of TMP/SMX monotherapy and a TMP/SMX Naranjo score of 9 strongly indicated this drug represented the most likely cause of her hyponatremia, though coadministration of several other medications known to cause hyponatremia may have compounded the severity of hyponatremia.

### 4.3 Contemporaneous hyponatremia and hyperkalemia in patients receiving TMP/SMX monotherapy

Hyperkalemia represents a well-characterized adverse effect of TMP/SMX. Hyperkalemia in patients developing TMP/SMX-induced hyponatremia may favor a direct drug effect upon the nephron, and disfavor SIADH, as the chief culprit generative intermediary mechanism. Shishido and colleagues (2012)^[19]^ describe a 76-year-old gentleman who underwent surgical resection of adenocarcinoma of the sigmoid colon. The patient subseuqnetly developed crescentic glomerulonephritis and appropriately received treatment with pulse high-dose corticosteroids and TMP/SMX chemoprophylaxis to prevent the onset of *P jiroveci* pneumonia. The patient coordinately developed de novo reductions of serum sodium and potassium concentration. Similarly, in a series of 7 patients receiving TMP/SMX monotherapy to treat *P jiroveci* pneumonia, 5 patients developed hyponatremia and all developed hyperkalemia.^[20]^ Khow and colleagues (2014)^[21]^ describe a series of 3 patients developing symptomatic hyponatremia following institution of TMP/SMX monotherapy, all of whom coordinately developed type 4 renal tubular normal anion gap acidosis and hyperkalemia. The coordinate development of hyponatremia and hyperkalemia following institution of TMP/SMX monotherapy may represent amiloride-like trimethoprim blockade of epithelial sodium channels (ENaCs), TMP/SMX-mediated blockade of aldosterone effect on nephrons, or yet to be determined effects of the compound on the proximal nephron.

### 4.4 Sodium and water homeostasis: clinically determining etiology of hyponatremia

An intimate understanding of mechanisms regulating total body sodium and total body water and a precise interpretation of appropriately obtained and available clinical and laboratory data prove fundamental and key in generating a mechanistic etiologic differential, and determining the most likely cause, explaining drug-induced iatrogenic hyponatremia. Pathophysiologic designation permits the internist, nephrologist, or intensivist to institute the most mechanistically-prudent pharmacological therapy.^[22]^ Serum sodium concentration represents a biochemical indicator of total body water and volume status determined by the physical examination represents a clinical indicator of total body sodium. This represents a key, and unfortunately frequently misunderstood, concept in clinical practice. Hyponatremia may be designated as hypertonic (eg, hyperglycemia, uremia, mannitol), isotonic (eg, hyperlipidemia, hyperproteinemia), or hypotonic (most common cause). Clinically determined volume status classifies hypotonic hyponatremia into hypervolemic, euvolemic, and hypovolemic types. The algorithm permits generation of a reasonable differential of the possible culprit diagnostic entities and the selection of a most likely causative diagnosis according to prudently performed physical examination, serum data, and urine chemistries.

### 4.5 Correlative relationship between TMP/SMX monotherapy and hyponatremia

Authors have extensively reported patients developing hyponatremia in the context of receiving TMP/SMX therapy, often recurring with treatment.^[6,7,10,13,22]^ Causality between TMP/SMX and hyponatremia may be presumed, though frequently remains uncertain^[6,13]^ The Naranjo score may be used to determine the probability of any given drug specifically precipitating an adverse effect.^[23]^ In a widely cited study conducted by Tsapepas et al,^[24]^ treatment with a high-dose of TMP/SMX (8 mg/kg/d) equal to or exceeding a duration of 3 days precipitated statistically significant reductions of the serum sodium concentration below the lower limit of normal (ie, 2 standard deviations below the population central tendency)in approximately three-fourths of individuals, with a decrease of the mean serum sodium from ∼138 to ∼132 mEq/L. Mean urine sodium concentrations averaged around ∼105 mEq/L. Severity of hyponatremia correlated with both total dose and duration of TMP/SMX therapy. Since the study utilized medical comorbidities or use of drugs causing hyponatremia as exclusionary criteria, We believe the overall prevalence and severity of TMP/SMX-induced hyponatremia in hospitalized patients was likely underestimated by this study, since the presence of medical conditions causing, or use of medications known to precipitate, hyponatremia represented exclusionary criteria to enhance the specificity of results and derivative interpretations.^[7,25]^ Standardly utilized doses of TMP/SMX may also cause hyponatremia, often more common in patients with glomerular or nephron-tubular dysfunction (85 vs 17%) and individuals receiving higher doses of the medication.^[4]^

### 4.6 Mechanisms underlying TMP/SMX-induced hyponatremia

Authors have posited renal salt wasting or the SIADH secretion to represent the chief intermediary mechanisms mediating TMP/SMX-induced hyponatremia (Table 3). Though fundamentally distinct pathogenically and pathophysiologically, these entities may share several clinical findings in common, including low serum uric acid, and/or low serum potassium and high urine osmolarity and urine sodium.^[9,26]^ Urine sodium and fractional excretion of sodium represent distinguishing laboratory findings consistent with renal salt wasting and urine sodium represents a distinguishing laboratory finding consistent with SIADH. Patients with SIADH experience suppressed proximal tubular reabsorption of sodium, secondary to high hydrostatic pressure in the peritubular capillary bed, patho-mechanistically distinguished proximal nephron-microvascular physiology from patients with renal salt wasting, who conversely exhibit low pericapillary hydrostatic pressures and enhanced proximal tubular reabsorption of sodium. Clinically-determined volume status readily distinguishes renal salt wasting from SIADH. Blood urea nitrogen and renin/aldosterone tend to be suppressed in SIADH and elevated in renal salt wasting.^[9]^ Differential response to different therapeutic modalities may be used as corollary evidence to patho-mechanistically identify the most likely culprit intermediary etiology generating TMP/SMX-induced hyponatremia. The trimethoprim component of Bactrim shares structural homology with the potassium-sparing diuretic distal convoluted tubule epithelial sodium channel antagonist amiloride.^[4,15,16,27–31]^

**Table 3:**
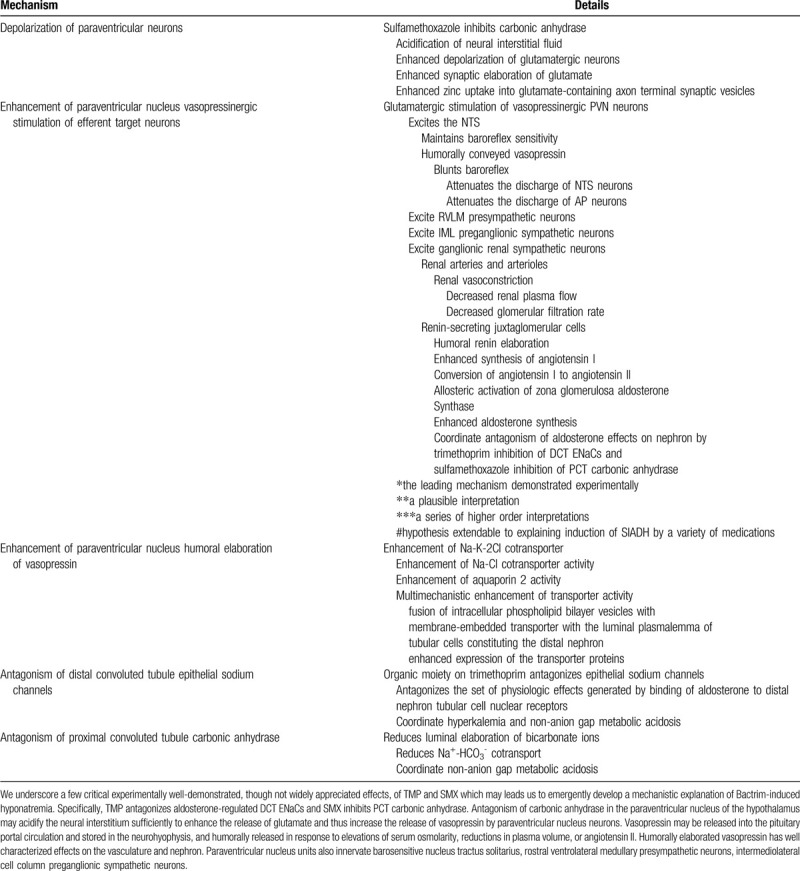
Bio-organic explanation of mechanisms underlying trimethoprim-sulfamethoxazole induced hyponatremia.

Trimethoprim antagonism of the epithelial sodium channel of the distal convoluted tubule may mechanistically contribute to TMP/SMX-induced hyponatremia.^[9,11,21,25,32]^ TMP/SMX antagonizes the molecular effects of aldosterone on the distal nephron,^[33]^ a mechanism consistent with the concurrent development of hyperkalemia and hyponatremia in patients receiving the medication; coadministration of TMP/SMX and corticosteroid therapy coordinately enhances the risk of developing hyperkalemia and its severity should it develop.^[34]^ Aldosterone receptor antagonism and hyperkalemia-induced inhibition of ammoniagenesis by the proximal convoluted tubule coordinately precipitate a non-anion gap metabolic acidosis complicated hypotonic hyponatremia. Several lines of evidence indicate a renal salt wasting mechanism for TMP/SMX-induced hyponatremia: High salt excretion in rats receiving trimethoprim, correlation between TMP/SMX dose and severity of reductions of hyponatremia, resolution of TMP/SMX-induced hyponatremia with intravenous salt repletion,^[9,14]^ and reversibility of TMP/SMX-induced hyponatremia with drug discontinuation would seem to indicate renal salt wasting represent the chief intermediary mechanism mediating reductions of the serum sodium concentration following treatment with TMP/SMX.^[14]^ Increases of the serum sodium in response to treatment with salt tabs and intravenous saline resuscitation, but to neither fluid restriction nor vasopressin receptor antagonists supports renal salt wasting, and disfavors SIADH, to represent the principal underlying mechanism of TMP/SMX-induced hyponatremia.^[9,11,21,25,32]^ The majority of cases of TMP/SMX-induced hyponatremia in the literature are consistent with renal salt wasting as the chief intermediary mechanism.

### 4.7 Perspectives and significance

We have accordingly provided the interested and distinguished reader with a brief discussion of the diagnostic considerations in identifying a culprit etiology of hyponatremia^[35,36]^ and a comprehensive evaluation of the literature seeking to determine the precise mechanisms by which TMP/SMX reduces serum sodium concentrations in human patients. SIADH and enhanced natriuresis represent parsimonious and plausible mechanisms explaining the observed set of effects, though a coherent model physio-mechanistically detailing the development of hyponatremia in the setting of TMP/SMX monotherapy remains to be more thoroughly elucidated. We discuss and detail the following effects extensively since sulfamethoxazole may plausibly enhance the hypothalamic release of vasopressin,^[37]^ which may powerfully and multi-mechanistically modulate total body water and sodium homeostasis (Table 4).^[38]^ Serum osmolarity, volume, and serum renin regulate the release of the pleiotropically-active nonapeptide vasopressin, which exerts a panoply of neural, neurohumoral, and renal effects from neurons of the paraventricular and supraoptic nuclei. Enhancement of serum osmolarity and renal renin release and reductions of effective arterial blood volume augment, and reductions of serum osmolarity and renal renin release and increases of effective arterial blood volume attenuate, the neuronal release of vasopressin.^[38]^ The unconventionally named exoenzyme renin cleaves hepatically synthesized angiotensinogen to angiotensin I, which undergoes cleavage to angiotensin II by angiotensin-converting enzyme studding the pulmonary vasculature.^[38]^ Angiotensin II enhances thirst, promotes preferential constriction of the efferent arteriole, generates balanced arterioconstriction and venoconstriction, and promotes the release of vasopressin from neurons of the paraventricular and supraoptic nuclei of the hypothalamus.^[38–40]^ Angiotensin II further undergoes cleavage to angiotensin III, the physiologic effects of which are similar, though somewhat less potent, compared with its precursor peptide.^[38–40]^

**Table 4:**
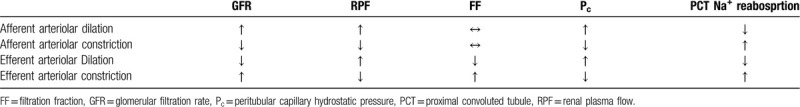
Renal arteriolar hemodynamics.

Neuronally-derived presynaptically-elaborated vasopressin conveys excitatory peptidergic synaptic drive to rostral ventrolateral medullary presympathetic,^[41,42]^ intermediolateral cell column preganglionic sympathetic,^[41,42]^ and barosensitive and chemosensitive nucleus tractus solitarius neurons,^[43]^ the former effects collectively enhancing sympathetic drive to the heart and vasculature and the latter effect sustaining baroreflex sensitivity. Dorsal medullo-hypothalamic projections from barosensitive nucleus tractus solitarius neurons may reciprocally tonically attenuate the elaboration of vasopressin.^[44]^ Ventral medullo-hypothalamic projections from neurons of the medullary lateral tegmental field (nucleus reticularis parvocellularis) neurons may synergistically enhance the release of vasopressin and excitatory peptidergic synaptic drive conveyed to rostral ventrolateral medullary presympathetic and intermediolateral cell column preganglionic sympathetic neurons.^[45]^ Vasopressin (and angiotensin II) humorally conveyed to nucleus tractus solitarius or area postrema neurons via the blood or cerebrospinal fluid blunts the baroreflex.^[46–48]^ Vasopressin ligand-effector coupling to G_q_-coupled vascular smooth muscle V1_a_ and V1_b_ receptors generates arterioconstriction and venoconstriction.^[39]^ Vasopressin ligand effector coupling to G_s_-coupled distal nephron V_2_ receptors enhances the expression, membrane insertion, and activity of phosphoregulated Na^+^-K^+^-2Cl^-^ cotransporters in the thick ascending limb of the loop of Henle, Na^+^-Cl^-^ cotransporters in the distal convoluted tubule, and aquaporin 2 channels in the collecting duct.^[40]^ These effects are mediated through fusion of intracellular phospholipid bilayer vesicles with membrane-embedded transporter with the luminal plasmalemma of tubular cells constituting the distal nephron and enhanced expression of the transporter proteins.^[40]^

Sulfamethoxazole inhibits intra-neuraxial^[37]^ and proximal convoluted tubule carbonic anhydrase.^[37]^ The latter effect reduces the enzymatic generation of bicarbonate ions luminally available to bind sodium ions, blunting the reabsorption of sodium ions and nephric capacity to augment effective arterial blood volume.^[49]^ The former effect promotes acidification of the neural interstitial milieu, enhancing the presynaptic and axon terminal vesicular uptake of the divalent cation zinc, co-released with glutamate.^[50]^ Sulfamethoxazole-mediated neural interstitial acidification and enhanced release of glutamate may enhance the release of vasopressin by the hypothalamus.^[37]^ Zinc may putatively substitute the divalent cation magnesium and antagonize synaptic and extra-synaptic NMDA glutamate receptors and attenuate sulfamethoxazole-mediated potentiation of glutamate receptor modulated signaling.^[50]^ We suggest at traditionally utilized doses, enhanced co-release of zinc sufficiently buffers the co-release of glutamate and mitigates putative sulfamethoxazole-induced amplification of vasopressin release. At higher doses, the presynaptic elaboration of glutamate exceeds the capacity of zinc to coordinately antagonize NMDA receptors, leaving sulfamethoxazole-induced amplification of vasopressin release unmitigated.^[4,9,17,30,33]^ We suggest pathways sharing mechanistic nodes in common with the foregoingly described set of effects may represent chief intermediary mechanisms generating drug-induced SIADH.^[36,37,40]^ We present these considerations conjecturally and subject them to the experimental interrogative efforts of Eminent internists, nephrologists, intensivists, and neurophysiologists.

### 4.8 Regulation of renal microvascular blood flow

Dynamic effects of sympathetic nerves and circulating mediators on renal blood flow^[51–53]^ may convergently modify proximal convoluted tubular reabsorption of sodium^[54]^ and prove culprit in drug-induced hyponatremia. Sympathetic nerve fibers innervate vascular smooth muscle cells of renal arteries and arterioles and renin-secreting cells of the juxtaglomerular apparatus.^[52]^ Experimental stimulation of distal transected ends of the renal sympathetic nerve generate renal vasoconstriction, enhance renin release, and reduce glomerular filtration rate and renal plasma flow.^[52]^ We tabularly summarize the effects of afferent and/or efferent arteriolar constriction or dilation upon glomerular filtration rate, renal plasma flow, filtration fraction, peritubular capillary hydrostatic pressure, and proximal convoluted tubular reabsorption of sodium.^[55]^ Constriction of either the afferent or efferent arteriole achieves potent reduction of peritubular capillary hydrostatic pressure, favoring reabsorption of sodium, with filtration fraction insignificantly modified.^[55]^ Dilation of either the afferent or efferent arteriole achieves potent augmentation of peritubular capillary hydrostatic pressure, blunting the reabsorption of sodium.^[56]^ We accordingly deduce coordinate dilatation of the afferent and efferent arterioles maintains a relatively constant proportionality between glomerular filtration rate and renal plasma flow and generates profound enhancement of tubular blood flow and peritubular capillary hydrostatic pressure, resisting proximal convoluted tubular reabsorption of sodium.^[56]^ Reciprocally, coordinate constriction of the afferent and efferent arterioles maintains a relatively constant proportionality between glomerular filtration rate and renal plasma flow and generates profound reductions of tubular blood flow and peritubular capillary hydrostatic pressure, enhancing proximal convoluted tubular reabsorption of sodium.^[56]^ Atrial natriuretic peptide, elaborated by significant stretch placed on the atrial walls, disfavors the reabsorption of anion-complexed sodium salts by the proximal convoluted tubule and promotes natriuresis.^[57]^

## 5 Conclusions

Hyponatremia represents an important and under recognized adverse effect of TMP/SMX. TMP/SMX therapy-induced hyponatremia may be more severe and prolonged in patients suffering from medical conditions or medications known to cause, or correlate with, reductions of the serum sodium concentration.^[58]^ The majority of investigators clinically describing TMP/SMX-induced hyponatremia provide evidence indicating nephrogenic salt wasting to represent the chief culprit intermediary culprit mechanism causing the condition.^[59]^ Conversely, we present a case report with clinical and laboratory features consistent with an SIADH-like syndrome precipitating TMP/SMX-induced worsening of a mild hyponatremia extant at initial admission. TMP/SMX-induced hyponatremia may be managed with drug discontinuation, intravenous saline hydration, high salt diet, or high-dose vasopressin receptor antagonist therapy. Underlying mechanism dictates therapeutic paradigm most likely to hasten clinical resolution. We present evidence that massive dose escalations of tolvaptan may effectively treat TMP/SMX-induced or TMP/SMX-accentuated hyponatremia in patients refractory to salt tablets, free water restriction, and traditionally used doses of vasopressin receptor antagonist therapy. Our findings may be extended to patients developing drug-induced hyponatremia with an SIADH-like syndrome (excessive secretion of ADH or excessive responsivity of distal convoluted and collecting tubule to ADH [increased receptor expression or enhanced ligand binding effector coupling]) In patients requiring continued treatment with TMP/SMX (those with a highly pathogenic multidrug resistance organism), intravenous saline hydration may effectively ameliorate or reverse the hyponatremia *sans* discontinuation of the drug.

## Acknowledgments

The first author dedicates the present treatise to Dr. Odette Annable, with whom he physically consummated emotional bonds frequently successively succeeding an inaugural courtship and the good people of Scandinavia and the Americas. The second author extends laudable heartfelt appreciation to his wife and offspring for their unfailing love and support.

## Author contributions

**Conceptualization:** Marc J. Kim.

**Data curation:** Marc J. Kim.

**Formal analysis:** Michael George Zaki Ghali, Marc J. Kim.

**Funding acquisition:** Marc J. Kim.

**Investigation:** Michael George Zaki Ghali, Marc J. Kim.

**Methodology:** Michael George Zaki Ghali, Marc J. Kim.

**Project administration:** Marc J. Kim.

**Resources:** Marc J. Kim.

**Supervision:** Marc J. Kim.

**Validation:** Marc J. Kim.

**Visualization:** Marc J. Kim.

**Writing – original draft:** Michael George Zaki Ghali, Marc J. Kim.

**Writing – review & editing:** Michael George Zaki Ghali, Marc J. Kim.
